# Leveraging 3D Atrial Geometry for the Evaluation of Atrial Fibrillation: A Comprehensive Review

**DOI:** 10.3390/jcm13154442

**Published:** 2024-07-29

**Authors:** Alexander J. Sharp, Timothy R. Betts, Abhirup Banerjee

**Affiliations:** 1 Institute of Biomedical Engineering, Department of Engineering Science, University of Oxford, Oxford OX3 7DQ, UK; 2 Cardiology Department, Oxford University Hospitals NHS Foundation Trust, Oxford OX3 9DU, UK; 3 Division of Cardiovascular Medicine, Radcliffe Department of Medicine, University of Oxford, Oxford OX3 9DU, UK

**Keywords:** atrial fibrillation, atrial cardiomyopathy, atrial geometry, three-dimensional imaging, statistical shape modeling, asymmetry index, left atrial sphericity, catheter ablation, stroke risk, personalized medicine

## Abstract

Atrial fibrillation (AF) is the most common sustained cardiac arrhythmia associated with significant morbidity and mortality. Managing risk of stroke and AF burden are pillars of AF management. Atrial geometry has long been recognized as a useful measure in achieving these goals. However, traditional diagnostic approaches often overlook the complex spatial dynamics of the atria. This review explores the emerging role of three-dimensional (3D) atrial geometry in the evaluation and management of AF. Advancements in imaging technologies and computational modeling have enabled detailed reconstructions of atrial anatomy, providing insights into the pathophysiology of AF that were previously unattainable. We examine current methodologies for interpreting 3D atrial data, including qualitative, basic quantitative, global quantitative, and statistical shape modeling approaches. We discuss their integration into clinical practice, highlighting potential benefits such as personalized treatment strategies, improved outcome prediction, and informed treatment approaches. Additionally, we discuss the challenges and limitations associated with current approaches, including technical constraints and variable interpretations, and propose future directions for research and clinical applications. This comprehensive review underscores the transformative potential of leveraging 3D atrial geometry in the evaluation and management of AF, advocating for its broader adoption in clinical practice.

## 1. Introduction

Atrial fibrillation (AF) is the most common sustained cardiac arrhythmia in adults, with a one in three lifetime risk of developing AF for individuals of European ancestry at age 55 years [1,2,3]. Global prevalence increased from approximately 33 million in 2010, to 50 million in 2020, with this rise expected to continue; this increase is multifactorial in nature, being associated with improved AF detection, life expectancy, and prevalence of comorbidities associated with AF (hypertension, diabetes mellitus, obesity, and obstructive sleep apnea) [1,2,4]. AF causes significant morbidity and mortality, representing a burden to both patients and health care economies, with more than 50% of AF patients having an impaired quality of life and a 10–40% annual hospitalization rate [1,2]. It is associated with a 1.5- to 3.5-fold increased risk of death and multiple adverse outcomes, including stroke, heart failure, cognitive impairment, myocardial infarction, sudden cardiac death, chronic kidney disease, and peripheral artery disease [1,2]. Both European and American guidelines describe three key elements in the management of AF: (1) managing risk of stroke; (2) symptom control, including managing AF burden through rhythm and rate control strategies; and (3) optimization of modifiable risk factors and addressing comorbidities [1,2].

The initial diagnostic evaluation of AF includes a comprehensive medical history, considering the aforementioned risk factors and concomitant conditions, in addition to those not previously mentioned, such as valvular heart disease, endocrine disorders, and infiltrative cardiomyopathies [1,2,5]. Subsequently, standard investigations include a 12-lead ECG to confirm the presence of both AF and ventricular rate, alongside assessing for the existence of conduction defects, ischemia, and structural heart disease; laboratory tests including full blood count, electrolytes, kidney, and thyroid function; and a transthoracic echocardiogram (TTE) to assess chamber size and function, valvular function, right ventricular pressure, and strain to detect underlying infiltrative cardiomyopathy [1,2]. Whilst not routine, depending on a patient’s signs or symptoms, additional imaging may be considered in the form of transesophageal echocardiography to assess valvular heart disease and for the presence of left atrial appendage (LAA) thrombus, computed tomography (CT) angiography for ischemia, and late gadolinium enhanced cardiac magnetic resonance imaging (MRI) to further guide AF treatment decisions [1,2].

All of these imaging modalities provide information on left atrial (LA) geometry, and the clinical utility of this has long been recognized. Two-dimensional (2D) approaches have utilized TTE in risk stratification of cardioembolic stroke [6] and predicting success of electrical cardioversion [7] or catheter ablation therapy with pulmonary vein isolation (PVI) [8]. However, 2D approaches have limitations due to their reliance on geometric assumptions; for example, echocardiographic measurement of LA diameter can underestimate the true LA size as it assumes a spherical shape [9,10]. Atrial remodeling is asymmetrical in nature, as highlighted by Cozma et al. [11] in 2007, who observed that with larger atria, there was a relatively greater increase in the LA basal than apical dimensions; this was termed trapezoidal remodeling. Whilst this can in part be accounted for by utilizing 2D measures of atrial area, true appreciation of changes in atrial geometry due to AF requires three-dimensional (3D) imaging modalities. High-resolution CT, MRI, and 3D echocardiography have paved the way for more accurate assessment of the atria’s asymmetrical 3D structure [9,10,12].

By leveraging 3D atrial geometry, we can improve the evaluation and management of AF, addressing existing knowledge gaps and enhancing patient outcomes. Given the significant advancements in this field and their impact on understanding the complex geometry of atria, there is need for a comprehensive review article that synthesizes current knowledge and identifies future research directions. This review aims to consolidate findings from recent studies, examining the advancements and challenges in LA geometry assessment to provide valuable insights for clinicians and researchers.

In this comprehensive review,

1. We discuss the interplay between 3D atrial geometry, atrial cardiomyopathy, and susceptibility to AF.
2. We explore the use of 3D atrial geometry in the assessment and treatment of AF, focusing on its use in stroke and rhythm management. We first discuss qualitative and simple quantitative metrics, before covering global quantitative metrics, and finally statistical shape modeling (SSM) approaches, covering measurement techniques and their clinical applications.
3. We provide insights into the limitations of current technologies and latest research directions, including deep learning-based SSM and spatiotemporal SSM, in this exciting and evolving field.

## 2. Atrial Cardiomyopathy and Its Impact on Atrial Geometry 

Atrial cardiomyopathy describes the negative remodeling which occurs due to AF. It has long been appreciated that “AF begets AF” [13,14,15]; sustained AF causes structural and functional changes within the atria that promote AF progression [16,17,18]. Experimental evidence since the 1990s has demonstrated atrial dilatation to be one such structural change [19]. The multiple wavelet hypothesis proposed by Moe [20,21] explains why dilated atria are more prone to AF. It considers fibrillation to be the result of the random propagation of multiple, independent electrical wavelets throughout the atria. These wavelets are generated by fractionation of the wavefront as it passes through tissue which is inhomogeneous with respect to excitability, refractory period, and conduction velocity; wavelets are self-sustaining and independent of the initial stimulus [17,18,22,23,24,25]. Dilated atria are more susceptible to maintaining AF, as the larger tissue mass supports a greater number of wavelets and the probability of them coalescing decreases [26].

In contrast, the hierarchical model of AF describes a degree of organization in AF, with distinct driver sites and disorganized propagation away from these sites. Driver sites may exhibit re-entrant, rotational, or focal activity, but they exist due to changes in the local functional and/or structural properties within the atria [17,18,27,28,29]. This has led to multiple studies investigating how these driver sites may be identified; these studies have demonstrated atrial cardiomyopathy to be a non-uniform process, both in regard to structural changes as identified by fibrosis formation using late gadolinium enhancement (LGE) on MRI [30,31,32,33] as well as areas of low voltage on electroanatomic voltage mapping (EAVM) [33,34,35,36] and functional changes as identified by areas of slow conduction velocity [16,33,37,38,39] as well as propagation patterns representing AF drivers [40,41,42]. Recognizing that atrial cardiomyopathy is not simply a global process gives us an appreciation of why 3D atrial geometry is required to truly understand geometric remodeling, considering not only atrial size, but also shape. 

## 3. Qualitative and Basic Quantitative Approaches for Understanding Atrial Shape

**Figure 1:**
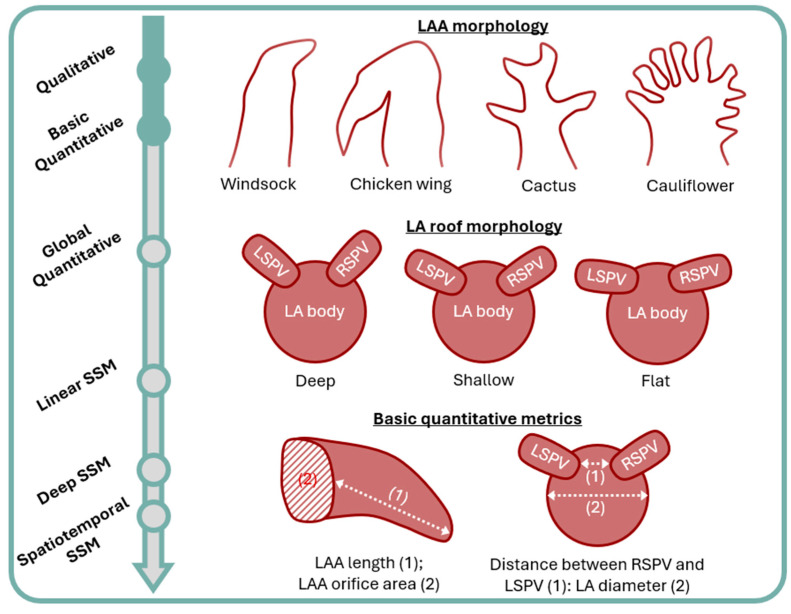
Overview of the different qualitative and basic quantitative metrics based on three-dimensional atrial geometry, used for the assessment of atrial fibrillation. LA: left atrium; LAA: left atrial appendage; LSPV: left superior pulmonary vein; RSPV: right superior pulmonary vein; SSM: statistical shape modeling.

Shape can be defined as the geometric information that describes an object irrespective of its pose; pose refers to the location, scale, and rotation of an object [43]. Geometric morphometrics combines descriptions of shape with statistical analyses describing patterns of shape variation [44]. Qualitative methods represent the simplest approach to describing elements of atrial shapes based on subjective morphological descriptions. These can be expanded through the inclusion of basic qualitative measures, such as measuring length and width of atrial sub-structures (Figure 1). 

In practice, such approaches have been applied to predicting stroke risk based on LAA morphology, which is considered a frequent source of thrombus formation and systemic embolization [45,46]. Both CT [45,46,47,48,49,50]- and MRI [47]-based approaches have been utilized, with the LAA categorized as either ‘chicken wing’, ‘windsock’, ‘cauliflower’, or ‘cactus’ in shape [45,47,48,49]. In addition, the anatomical relationship between the LAA and left superior pulmonary vein has been described as either high-, mid-, or low-type [45], and the LAA ostium as oval, foot, triangular, water drop, or round [45]. Basic quantitative measurements include LAA volume, length, and LAA orifice area [46,50]. However, results of these studies can be contradictory, with both ‘chicken wing’ [47] and ‘windsock’ [48] morphologies being less frequently observed in patients with a history of stroke or transient ischemic attack (TIA) and with LAA ostium area found both useful [46] and not [50] as a predictive marker. A key issue of this approach is that LAA morphology is incredibly diverse, making classification into pre-defined categories challenging and prone to interobserver variation [45,51,52].

Regarding AF burden and rhythm control, the LA roof has been classified as either deep, shallow, or flat in shape, depending on the angle of insertion of the superior pulmonary veins to the LA body. A flat roof was associated with more significant atrial cardiomyopathy in a study by Kurotobi et al. [53], which demonstrated more persistent AF (perAF), larger LA volumes (LAV), larger LA diameters, and more burst inducible atrial tachycardia after PVI in this cohort. Chen et al. [54] investigated the ratio of distance between the right and left superior pulmonary veins and LA diameter, showing it to be an independent predictor of AF recurrence post PVI in a multivariable analysis. Kim et al. [55] assessed the impact of a narrow or wide left lateral ridge, in a multivariate analysis showing narrow ridges to be an independent predictor of recurrence. Interestingly, a follow-up experimental study using porcine atrial tissue demonstrated contact force and radio-frequency lesion formation to be significantly lower on narrow vs. wide ridges, implying recurrence in patients with this shape variation may not only be related to cardiomyopathy but our ability to deliver successful ablation treatment. Sorgente et al. [56] also investigated ablation technique, in this instance finding cryoballoon occlusion to be worse in patients with shallower angles between the LA body and pulmonary veins. 

## 4. Global Quantitative Approaches

**Figure 2:**
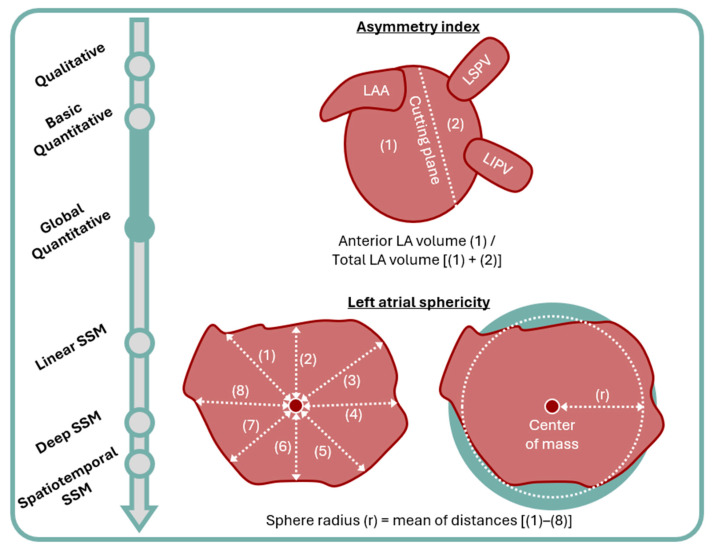
Overview of the asymmetry index and left atrial sphericity measurement. LA: left atrium; LAA: left atrial appendage; LIPV: left inferior pulmonary vein; LSPV: left superior pulmonary vein; SSM: statistical shape modeling.

Atrial asymmetry index (ASI) and left atrial sphericity (LASP) (Figure 2) are the two most well-described global quantitative approaches for understanding atrial shapes. ASI [57] is a ratio of the anterior LA to total LA volume. It is calculated from 3D reconstructions of the LA, by dividing it into anterior and posterior segments via a cutting plane that runs parallel to the posterior wall, between the pulmonary vein ostia and LAA. Preferential anterior dilatation in AF is hypothesized to be the result of the physical constraints imposed by the spine. Pathophysiologically, the interplay of ASI and mechanical remodeling has been explored in studies utilizing both CT and echocardiographic imaging; these studies have shown higher asymmetry index to be associated with diastolic dysfunction and delayed anterior mechanical activation [58,59]. Furthermore, exploring the interplay of LAV and ASI, Nedios et al. [57] demonstrated increases in LAV and ASI in AF patients vs. healthy controls but, interestingly, only higher LAVs in paroxysmal (pAF) vs. perAF patients and only higher ASI in persistent vs. longstanding perAF patients; both LAV and ASI were shown to be independent predictors of AF recurrence post PVI. These results highlight the multifactorial nature of geometric remodeling in AF and the potential benefits of utilizing both in assessing patients. In contrast, Guo et al. [60] found ASI not to be an independent predictor of AF recurrence following catheter ablation. This study did not distinguish between persistent and longstanding perAF, which may in part explain these differing results. 

LASP [61] is calculated as the variation between LA shape and a best fit sphere. The sphere’s radius is calculated as the mean distance between all points on the LA wall and its center of mass. The coefficient of variation is calculated as the ratio of standard deviation in this mean distance measurement and the mean radius. Volume overload is a key component of atrial cardiomyopathy. A sphere has the smallest surface area to volume ratio of all 3D shapes, as such spherical remodeling is hypothesized to occur because it represents the optimal geometrical adaptation for minimizing wall stress [61]. LASP has been applied to predicting stroke risk, where it was shown to outperform CHA_2_DS_2_-VASc scoring in a population of AF patients with a history of previous thromboembolic events, compared to the age- and sex-matched controls [62]. Primarily, however, studies have applied LASP to managing AF burden. Here, it has been shown to be an independent predictor of AF recurrence following external cardioversion [63] and catheter ablation [60,61,64,65]. Combining LASP with other independent predictors from these studies, such as LA minimal volume index [60] and clinical factors [65], may maximize its potential. Similarly, LASP has been shown to improve after successful catheter ablation [66]; for this use, combining it with LAV may serve as a better marker of response than LAV alone, as LAV may improve from ablation scar-related LA retraction rather than beneficial remodeling. Notwithstanding these encouraging results, a study by Mulder et al. [67] comparing AF and healthy control patients demonstrated no difference in LASP, despite differences in LA diameter, LAV, and LAV index. Likewise, Bossard et al. [68] did not find LASP to be an independent predictor of recurrence following catheter ablation of AF.

In interpreting these discrepancies, we should remember that cardiac anatomy is highly complex. Remodeled atria may have all the components of a flat LA roof, higher sphericity, and anterior dilatation, but clinical utilization of these measures requires establishing threshold values to separate abnormal from normal. Global measures may not be able to capture subtle changes [69]. Shape is complex and therefore requires a more complex analysis [70].

## 5. Statistical Shape Modeling

### 5.1. An Overview of Statistical Shape Modeling

**Figure 3:**
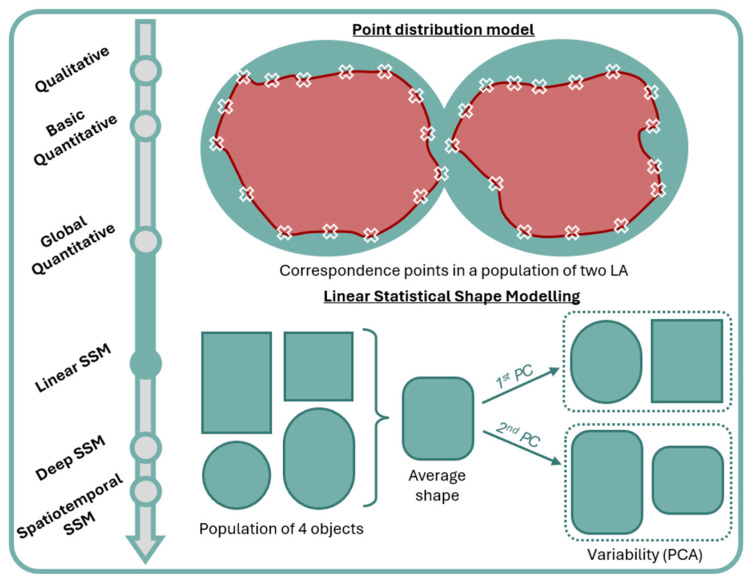
Correspondence points (white ‘X’ symbols) in a point distribution model and average shape and shape variability in a linear SSM. First PC captures the roundedness of edges and second PC captures the ratio of width to length. LA: left atrium; PC: principal component; PCA: principal component analysis; SSM: statistical shape modeling.

SSM is the computational extension of geometric morphometrics [71], providing quantitative descriptions of shape with excellent geometric detail and statistical power [72]. To produce statistical shape models, 3D anatomical information must first be converted into a discrete representation [73]. In medical imaging, this is most commonly achieved through the use of correspondence points (Figure 3); these are dense sets of points that represent 3D geometry and, importantly, whose location corresponds between objects in a population [74]. This type of SSM is called a point distribution model (PDM); it has the advantage, over a deformation field approach, of being easier to visualize and therefore interpret for clinicians as well as being less impacted by noise [73,75,76]. PDMs are the evolution of morphometric landmark approaches, not relying on a limited number of manually identified landmarks/correspondence points but instead consisting of dense sets of hundreds to thousands of correspondence points that are produced automatically, meaning geometry is modeled with greater fidelity [77,78]; as the position of the correspondence points cannot be determined manually, their appropriate location is extrapolated from the shapes themselves [79].

Subsequently, a sophisticated geometric model can be produced, which captures both the population average and variability [71,72,73,74,75] (Figure 3). Average geometry across a population is defined as the average of correspondence point positions [70]. A principal component analysis (PCA) is frequently utilized to describe variability. PCA is a multivariate statistical method which facilitates the interpretation of large datasets by reducing their dimensionality whilst maintaining information content [80,81]. A principal component (PC) is a linear function that combines information from multiple original variables to produce a new, single variable. A combination of uncorrelated PCs is sought, which maximally explains variance within all the original variables. The maximum possible number of PCs is the same as the number of original variables, and a combination of all possible PCs would explain all variance within a dataset. However, the aim of PCA is to reduce the dimensionality of a dataset by utilizing fewer PCs to aid interpretation and visualization of the original dataset. This is achieved by maximizing variance within the first PC, followed by maximizing remaining variance in the second PC and so forth, such that PCs with low information content can be disregarded. 

In PDMs, by selecting PCs which account for the majority of observed variance, each object can be described as a vector of scalar values with a dimensionality equal to the number of selected PCs; disregarding PCs accounting for the lower end of variance likely equates to excluding variance which is the result of noise from the image sampling process itself [43,77,80]. Each PC describes a mode of shape variation [70,71,73], and this facilitates better appreciation of anatomical differences, both on individual and population levels [76]. Furthermore, this approach gives SSM generative power, as Gaussian variation in the PCs can produce new and plausible objects, not contained in the original dataset [73,74]. SSM has been widely utilized for modeling the human heart [82,83,84], including in the context of AF.

### 5.2. Applications of Statistical Shape Models in Atrial Fibrillation

Cates et al. [77] utilized SSM of the LA and LAA from MRI, to compare cohorts of patients with and without spontaneous echo contrast (SEC) in the LAA. SEC is a sign of blood stasis and therefore considered a risk of thrombus formation and stroke. Of the variation, 95% was captured by the first two PCs, with them finding longer LAA length and anterior orientation relative to the LA to be associated with SEC. SSM of the LAA has also been shown to be able to capture clinically relevant variation, with an ability to divide patients into the traditional four morphological groups, mitigating its previously subjective nature [71]. Whilst neither of these studies directly associated geometry with stroke, subsequent studies have compared groups with a history of stroke or TIA with a control group. These have shown relative alignment of the LA and LAA to be a key component of stroke prediction [85], as well as the LAA being broader, shorter, and less angulated [52]; combining such shape parameters with CHA_2_DS_2_-VASc score improved prediction performance [52]. 

Using SSM to compare LA geometry in pAF, perAF, and control patients, Cates et al. [77] demonstrated that the majority of variation (21%) was captured by a PC which captured dilation in the antero-posterior direction; such variation could be considered similar to increased ASI and LASP. Multiple studies have used LA SSM in predicting recurrence following catheter ablation. By comparing local area changes in patients with and without recurrence, Jia et al. [86] derived a shape score with predictive value that outperformed traditional methods and, in combination with AF persistence and LAV, showed a high predictive value. Bieging et al. [70] demonstrated that LA shape was independent of even LA fibrosis as a predictor of AF recurrence, suggesting that both LA shape and fibrosis are important factors in atrial cardiomyopathy; in this study, recurrence was again associated with more spherical LA. Similarly, Varela et al. [87] found recurrent LA to be more spherical, with flattening at the roof.

A comprehensive understanding of atrial remodeling involves combining multiple different measures which assess its multiple elements, for example, LGE MRI for assessing fibrosis and EAVM for assessing voltage and conduction velocity. Different technologies produce variations in 3D geometry; as such, SSM has been used to produce average atrial geometries which can then be used as a basis to combine multiple measures onto a single geometry, minimizing variation due to spatial displacement. This has been applied in clinical studies assessing correlation between these variables [33,88]. Similarly, SSM has been a fundamental step in the creation of *in silico* digital twins used in catheter ablation of AF; here, they facilitate the integration of the multiple variables which contribute to arrhythmogenesis, combining individual patient data from LGE MRI and EAVM, with data on fiber orientation from existing atrial atlases [89,90,91]. By simulating different treatment strategies based on patient-specific variables, the optimal approach can be chosen for a patient [92,93]. A further use of SSM in the creation of digital twins is their potential to enable 3D whole-heart reconstruction, including both atria and ventricles, from a limited number of clinical cine MRI slices [94].

The generative power of SSM is of particular value in the development of machine learning classifiers *in silico*. Training of machine learning models requires large and balanced datasets with reliable labeling. SSM’s generative ability means an almost infinite ability to generate realistic geometries and subsequently datasets with ground truth labels [74]. This can significantly expand the size of training datasets compared to studies utilizing clinical data [12,95]. For example, in the personalization of catheter ablation therapy, running multiple simulations of the impact of different ablation strategies is computationally expensive, with long simulation times, thus limiting clinical feasibility. Zolotarev et al. [92] addressed this limitation by simulating responses to various treatments in 1000 virtual patients. These simulations were then used to train a deep learning model which can predict treatment response to different therapies based on anatomical and physiological features from only the pre-ablation atrial fibrillation simulations.

## 6. Challenges and Future Directions

**Figure 4:**
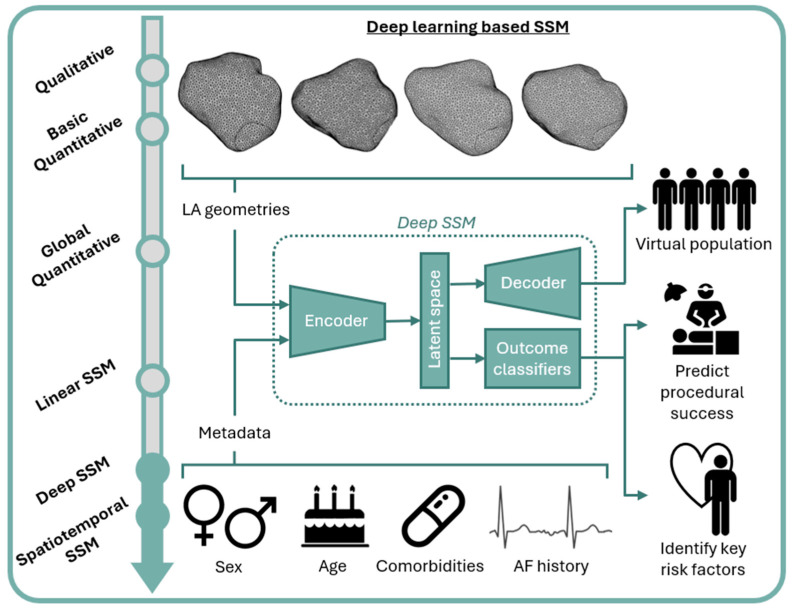
Deep learning-based SSM capturing population-wide LA geometries and metadata to produce virtual populations relevant to specified sub-populations, personalized prediction of procedural success, and identification of key risk factors. LA: left atrium; SSM: statistical shape modeling.

Traditional SSM exploring linear relationships such as PCA faces a key challenge in that variability in human anatomy may be non-linear. Deep learning-based SSM (Figure 4) may overcome this limitation, capturing population-wide anatomical variability [76]. An advantage of deep learning-based approaches is the existence of a latent space aiming to provide accurate low-dimensional and disentangled representation of the high-dimensional input data, where each latent dimension encodes a different aspect of the inter-subject variability. The non-linearities in the deep learning architecture enable the modeling of richer and more condensed relationships between high-dimensional input data and low-dimensional latent space representations [96,97,98]. 

A further advantage of this approach is the ability to include patient metadata such as age, sex, comorbidities, and AF clinical history in the model, which may greatly improve the ability of SSM to generate geometries relevant to specified sub-populations. Improved understanding of the impact of specific clinical characteristics on geometry may facilitate more targeted intervention [99]. Furthermore, such SSMs are generalizable to even under-represented patient cohorts in the original dataset [100,101]; this is a key consideration as we strive towards improved equality, diversity, and inclusion in research. An additional ethical concern is the potentially prohibitive cost and limited availability of imaging modalities such as MRI and CT, on which SSM is frequently reliant. To maximize the equitable distribution of scientific advancement, future work should seek to translate findings to alternative imaging modalities, for example, through deriving shape metrics which may then be applied to TTE [69].

This review has primarily focused on the role of atrial geometry in managing risk of stroke and AF burden; yet, the optimization of modifiable risk factors and addressing a patient’s comorbidities are key considerations in holistic management and an area where the role of atrial geometry is relatively unexplored.

Finally, we must consider that AF is not a static condition. There are temporal changes, in terms of dynamic atrial motion through the cardiac cycle, and also progression of atrial cardiomyopathy over time; spatiotemporal SSM [75,102,103,104] can capture this geometric variation. It may provide invaluable insights into the impact of atrial cardiomyopathy on mechanical function and improve the ability of computational fluid dynamics simulations [105,106] to model stroke risk based on dynamic LAA morphology. By tracking changes in atrial structure over time, these tools can help identify early signs of disease and monitor the efficacy of interventions, thus allowing for a more comprehensive understanding of the disease trajectory and facilitating personalized treatment strategies that can adapt to the patient’s evolving condition.

## 7. Conclusions

AF represents a significant and growing public health challenge, characterized by its complex interplay with various comorbidities and its substantial impact on morbidity and mortality. Three-dimensional imaging modalities have significantly advanced our understanding and management of AF, allowing for more accurate assessment of the atria’s asymmetrical structure. Whereas global quantitative approaches such as ASI and LASP benefit from their relative simplicity, SSM offers a more objective and powerful tool in the study of atrial geometry, providing detailed quantitative descriptions of shape and facilitating the integration of multiple variables affecting AF. By capturing both population averages and variability, SSM has improved our understanding of atrial remodeling and its clinical implications. Applications of SSM in assessing stroke risk, predicting AF recurrence, and personalizing catheter ablation therapy highlight its potential in enhancing patient-specific management strategies. Whilst traditional SSM may not fully capture the non-linear variability of human anatomy, advances in deep learning-based SSM offers promising solutions, which, in combination with spatiotemporal SSM’s ability to capture dynamic changes in atrial geometry over time, could lead to more precise and personalized treatment strategies for patients with AF. By continuing to refine these approaches and integrating new technologies, we can better address the complexities of AF and ultimately improve patient outcomes.

## Data Availability

Data sharing is not applicable to this article as no new data were created or analyzed in this study. Data discussed in this review are available in the original publications cited.

## References

[1] Hindricks G., Potpara T., Dagres N., Bax J.J., Boriani G., Dan G.A., Fauchier L., Kalman J.M., Lane D.A., Lettino M. (2021). 2020 ESC Guidelines for the Diagnosis and Management of Atrial Fibrillation Developed in Collaboration with the European Association for Cardio-Thoracic Surgery (EACTS). Eur. Heart J..

[2] Joglar J.A., Chung M.K., Armbruster A.L., Benjamin E.J., Chyou J.Y., Cronin E.M., Deswal A., Eckhardt L.L., Goldberger Z.D., Gopinathannair R. (2024). 2023 ACC/AHA/ACCP/HRS Guideline for the Diagnosis and Management of Atrial Fibrillation: A Report of the American College of Cardiology/American Heart Association Joint Committee on Clinical Practice Guidelines. J. Am. Coll. Cardiol..

[3] Staerk L., Wang B., Preis S.R., Larson M.G., Lubitz S.A., Ellinor P.T., McManus D.D., Ko D., Weng L.C., Lunetta K.L. (2018). Lifetime Risk of Atrial Fibrillation According to Optimal, Borderline, or Elevated Levels of Risk Factors: Cohort Study Based on Longitudinal Data from the Framingham Heart Study. BMJ.

[4] Chugh S.S., Havmoeller R., Narayanan K., Singh D., Rienstra M., Benjamin E.J., Gillum R.F., Kim Y.H., McAnulty J.H., Zheng Z.J. (2014). Worldwide Epidemiology of Atrial Fibrillation: A Global Burden of Disease 2010 Study. Circulation.

[5] Jaiswal V., Agrawal V., Khulbe Y., Hanif M., Huang H., Hameed M., Shrestha A.B., Perone F., Parikh C., Gomez S.I. (2023). Cardiac Amyloidosis and Aortic Stenosis: A State-of-the-Art Review. Eur. Hear. J. Open.

[6] Tan T.C., Nunes M.C.P., Handschumacher M., Pontes-Neto O., Park Y.-H., O’Brien C., Piro V., Kim G.-M., Helenius J., Zeng X. (2020). Left Atrial Cross-Sectional Area Is a Novel Measure of Atrial Shape Associated with Cardioembolic Strokes. Heart.

[7] Henry W.L., Morganroth J., Pearlman A.S., Clark C.E., Redwood D.R., Itscoitz S.B., Epstein S.E. (1976). Relation between Echocardiographically Determined Left Atrial Size and Atrial Fibrillation. Circulation.

[8] Berruezo A., Tamborero D., Mont L., Benito B., Tolosana J.M., Sitges M., Vidal B., Arriagada G., Méndez F., Matiello M. (2007). Pre-Procedural Predictors of Atrial Fibrillation Recurrence after Circumferential Pulmonary Vein Ablation. Eur. Heart J..

[9] Tops L.F., Schalij M.J., Bax J.J. (2010). Imaging and Atrial Fibrillation: The Role of Multimodality Imaging in Patient Evaluation and Management of Atrial Fibrillation. Eur. Heart J..

[10] Bisbal F., Guiu E., Calvo N., Marin D., Berruezo A., Arbelo E., Ortiz-Pérez J., De Caralt T.M., Tolosana J.M., Borràs R. (2013). Left Atrial Sphericity: A New Method to Assess Atrial Remodeling. Impact on the Outcome of Atrial Fibrillation Ablation. J. Cardiovasc. Electrophysiol..

[11] Cozma D., Popescu B.A., Lighezan D., Lucian P., Mornos C., Ginghina C., Dragulescu S.-I. (2007). Left Atrial Remodeling: Assessment of Size and Shape to Detect Vulnerability to Atrial Fibrillation. Pacing Clin. Electrophysiol..

[12] Firouznia M., Feeny A.K., Labarbera M.A., Mchale M., Cantlay C., Kalfas N., Schoenhagen P., Saliba W., Tchou P., Barnard J. (2021). Machine Learning—Derived Fractal Features Pulmonary Veins from Cardiac Computed Tomography Scans Are Associated With Risk of Recurrence of Atrial Fibrillation Postablation. Circ. Arrhythm. Electrophysiol..

[13] Wijffels M.C.E.F., Kirchhof C.J.H.J., Boersma L.V.A., Dorland R., Allessie M.A. (1993). Atrial Fibrillation Begets Atrial Fibrillation. New Trends Arrhythm..

[14] Li D., Fareh S., Ki Leung T., Nattel S. (1999). Promotion of Atrial Fibrillation by Heart Failure in Dogs Atrial Remodeling of a Different Sort. Circulation.

[15] Morillo C.A., Klein G.J., Jones D.L., Guiraudon C.M. (1995). Chronic Rapid Atrial Pacing. Circulation.

[16] Silva Garcia E., Lobo-Torres I., Fernández-Armenta J., Penela D., Fernandez-Garcia M., Gomez-Lopez A., Soto-Iglesias D., Fernández-Rivero R., Vazquez-Garcia R., Acosta J. (2023). Functional Mapping to Reveal Slow Conduction and Substrate Progression in Atrial Fibrillation. Europace.

[17] Roney C.H., Wit A.L., Peters N.S. (2019). Challenges Associated with Interpreting Mechanisms of AF. Arrhythm. Electrophysiol. Rev..

[18] Goette A., Kalman J.M., Aguinaga L., Akar J., Cabrera J.A., Chen S.A., Chugh S.S., Corradi D., D’Avila A., Dobrev D. (2017). EHRA/HRS/APHRS/SOLAECE Expert Consensus on Atrial Cardiomyopathies: Definition, Characterization, and Clinical Implication. Heart Rhythm.

[19] Sanfilippo A.J., Abascal V.M., Sheehan M., Oertel L.B., Harrigan P., Hughes R.A., Weyman A.E. (1990). Atrial Enlargement as a Consequence of Atrial Fibrillation A Prospective Echocardiographic Study. Circulation.

[20] Moe G.K., Rheinboldt W.C., Abildskov J.A. (1964). A Computer Model of Atrial Fibrillation. Am. Heart J..

[21] Moe G.K., Abildskov J.A. (1959). Atrial Fibrillation as a Self-Sustaining Arrhythmia Independent of Focal Discharge. Am. Heart J..

[22] Nishida K., Datino T., Macle L., Nattel S. (2014). Atrial Fibrillation Ablation: Translating Basic Mechanistic Insights to the Patient. J. Am. Coll. Cardiol..

[23] Dukkipati S.R., Reddy V.Y. (2017). Catheter Ablation of “Rotors” for the Treatment of AF: Should We Drink the Kool-Aid?. J. Am. Coll. Cardiol..

[24] Williams S.E., Linton N., O’Neill L., Harrison J., Whitaker J., Mukherjee R., Rinaldi C.A., Gill J., Niederer S., Wright M. (2017). The Effect of Activation Rate on Left Atrial Bipolar Voltage in Patients with Paroxysmal Atrial Fibrillation. J. Cardiovasc. Electrophysiol..

[25] van Staveren L.N., de Groot N.M.S. (2020). Exploring Refractoriness as an Adjunctive Electrical Biomarker for Staging of Atrial Fibrillation. J. Am. Heart Assoc..

[26] Garrey W. (1914). The Nature of Fibrillary Contraction of the Heart—Its Relation to Tissue Mass and Form. Am. J. Physiol..

[27] Pandit S.V., Jalife J. (2013). Rotors and the Dynamics of Cardiac Fibrillation. Circ. Res..

[28] Vaquero M., Calvo D., Jalife J. (2008). Cardiac Fibrillation: From Ion Channels to Rotors in the Human Heart. Heart Rhythm.

[29] Jansen H.J., Bohne L.J., Gillis A.M., Rose R.A. (2020). Atrial Remodeling and Atrial Fibrillation in Acquired Forms of Cardiovascular Disease. Heart Rhythm O2.

[30] Marrouche N.F., Wazni O., McGann C., Greene T., Dean J.M., Dagher L., Kholmovski E., Mansour M., Marchlinski F., Wilber D. (2022). Effect of MRI-Guided Fibrosis Ablation vs Conventional Catheter Ablation on Atrial Arrhythmia Recurrence in Patients with Persistent Atrial Fibrillation: The DECAAF II Randomized Clinical Trial. JAMA.

[31] Marrouche N.F., Wilber D., Hindricks G., Jais P., Akoum N., Marchlinski F., Kholmovski E., Burgon N., Hu N., Mont L. (2014). Association of Atrial Tissue Fibrosis Identified by Delayed Enhancement MRI and Atrial Fibrillation Catheter Ablation: The DECAAF Study. JAMA J. Am. Med. Assoc..

[32] Ohguchi S., Inden Y., Yanagisawa S., Fujita R., Yasuda K., Katagiri K., Oguri M., Murohara T. (2022). Regional Left Atrial Conduction Velocity in the Anterior Wall Is Associated with Clinical Recurrence of Atrial Fibrillation after Catheter Ablation: Efficacy in Combination with the Ipsilateral Low Voltage Area. BMC Cardiovasc. Disord..

[33] Nairn D., Eichenlaub M., Müller-Edenborn B., Huang T., Lehrmann H., Nagel C., Azzolin L., Luongo G., Figueras Ventura R.M., Rubio Forcada B. (2023). Differences in Atrial Substrate Localization Using LGE-MRI, Electrogram Voltage and Conduction Velocity—A Cohort Study Using a Consistent Anatomical Reference Frame in Patients with Persistent Atrial Fibrillation. Europace.

[34] Miyamoto K., Tsuchiya T., Narita S., Yamaguchi T., Nagamoto Y., Ando S.I., Hayashida K., Tanioka Y., Takahashi N. (2009). Bipolar Electrogram Amplitudes in the Left Atrium Are Related to Local Conduction Velocity in Patients with Atrial Fibrillation. Europace.

[35] Huo Y., Gaspar T., Schönbauer R., Wójcik M., Fiedler L., Roithinger F.X., Martinek M., Pürerfellner H., Kirstein B., Richter U. (2022). Low-Voltage Myocardium-Guided Ablation Trial of Persistent Atrial Fibrillation. NEJM Evid..

[36] Masuda M., Fujita M., Iida O., Okamoto S., Ishihara T., Nanto K., Kanda T., Tsujimura T., Matsuda Y., Okuno S. (2018). Left Atrial Low-Voltage Areas Predict Atrial Fibrillation Recurrence after Catheter Ablation in Patients with Paroxysmal Atrial Fibrillation. Int. J. Cardiol..

[37] Dang L., Angel N., Zhu M., Vesin J.M., Scharf C. (2022). Correlation between Conduction Velocity and Frequency Analysis in Patients with Atrial Fibrillation Using High-Density Charge Mapping. Med. Biol. Eng. Comput..

[38] Kishima H., Mine T., Fukuhara E., Takahashi S., Ishihara M. (2020). Is the Abnormal Conduction Zone of the Left Atrium a Precursor to a Low Voltage Area in Patients with Atrial Fibrillation?. J. Cardiovasc. Electrophysiol..

[39] Okubo Y., Oguri N., Sakai T., Uotani Y., Furutani M., Miyamoto S., Miyauchi S., Okamura S., Tokuyama T., Nakano Y. (2024). Conduction Velocity Mapping in Atrial Fibrillation Using Omnipolar Technology. PACE—Pacing Clin. Electrophysiol..

[40] Betts T.R., Good W.W., Melki L., Metzner A., Grace A., Verma A., Murray S., James S., Wong T., Boersma L.V.A. (2023). Treatment of Pathophysiologic Propagation Outside of the Pulmonary Veins in Retreatment of Atrial Fibrillation Patients: RECOVER AF Study. Europace.

[41] Pope M.T.B., Kuklik P., Briosa e Gala A., Leo M., Mahmoudi M., Paisey J., Betts T.R. (2021). Spatial and Temporal Variability of Rotational, Focal, and Irregular Activity: Practical Implications for Mapping of Atrial Fibrillation. J. Cardiovasc. Electrophysiol..

[42] Lee J.M.S., Nelson T.A., Clayton R.H., Kelland N.F. (2022). Characterization of Persistent Atrial Fibrillation with Non-Contact Charge Density Mapping and Relationship to Voltage. J. Arrhythm..

[43] Stegmann M.B., Delgado Gomez D. (2002). A Brief Introduction to Statistical Shape Analysis.

[44] Adams D.C., Rohlf F.J., Slice D.E. (2004). Geometric Morphometrics: Ten Years of Progress Following the ‘Revolution’. Ital. J. Zool..

[45] Wang Y., Di Biase L., Horton R.P., Nguyen T., Morhanty P., Natale A. (2010). Left Atrial Appendage Studied by Computed Tomography to Help Planning for Appendage Closure Device Placement. J. Cardiovasc. Electrophysiol..

[46] Lee J.M., Kim J.B., Uhm J.S., Pak H.N., Lee M.H., Joung B. (2017). Additional Value of Left Atrial Appendage Geometry and Hemodynamics When Considering Anticoagulation Strategy in Patients with Atrial Fibrillation with Low CHA2DS2-VASc Scores. Heart Rhythm.

[47] Di Biase L., Santangeli P., Anselmino M., Mohanty P., Salvetti I., Gili S., Horton R., Sanchez J.E., Bai R., Mohanty S. (2012). Does the Left Atrial Appendage Morphology Correlate with the Risk of Stroke in Patients with Atrial Fibrillation? Results from a Multicenter Study. J. Am. Coll. Cardiol..

[48] Simon J., Smit J.M., El Mahdiui M., Száraz L., van Rosendael A.R., Zsarnóczay E., Nagy A.I., Gellér L., van der Geest R.J., Bax J.J. (2024). Association of Left Atrial Appendage Morphology and Function with Stroke and Transient Ischemic Attack in Atrial Fibrillation Patients. Am. J. Cardiol..

[49] Kimura T., Takatsuki S., Inagawa K., Katsumata Y., Nishiyama T., Nishiyama N., Fukumoto K., Aizawa Y., Tanimoto Y., Tanimoto K. (2013). Anatomical Characteristics of the Left Atrial Appendage in Cardiogenic Stroke with Low CHADS2 Scores. Heart Rhythm.

[50] Dudzińska-Szczerba K., Michałowska I., Piotrowski R., Sikorska A., Paszkowska A., Stachnio U., Kowalik I., Kułakowski P., Baran J. (2021). Assessment of the Left Atrial Appendage Morphology in Patients after Ischemic Stroke—The ASSAM Study. Int. J. Cardiol..

[51] Shin S.Y., Park J.W. (2021). Is the Left Atrial Appendage (LAA) Anatomical Shape Really Meaningless Measure for Stroke Risk Assessment?. Int. J. Cardiol..

[52] Bieging E.T., Morris A., Chang L., Dagher L., Marrouche N.F., Cates J. (2021). Statistical Shape Analysis of the Left Atrial Appendage Predicts Stroke in Atrial Fibrillation. Int. J. Cardiovasc. Imaging.

[53] Kurotobi T., Iwakura K., Inoue K., Kimura R., Toyoshima Y., Ito N., Mizuno H., Shimada Y., Fujii K., Nanto S. (2011). The Significance of the Shape of the Left Atrial Roof as a Novel Index for Determining the Electrophysiological and Structural Characteristics in Patients with Atrial Fibrillation. Europace.

[54] Chen W.T., Chang S.L., Lin Y.J., Lo L.W., Hu Y.F., Chao T.F., Chung F.P., Liao J.N., Huang Y.C., Hsieh M.H. (2014). The Impact of Anatomical Remodeling of the Left Atrium and Pulmonary Vein on the Recurrence of Paroxysmal Atrial Fibrillation after Catheter Ablation. Int. J. Cardiol..

[55] Kim S., Kim Y.R., Nam G.B., Choi K.J., Kim Y.H. (2021). The Shape of the Left Lateral Ridge as a Predictor of Long-Term Outcome of Catheter Ablation for Atrial Fibrillation Based on Clinical and Experimental Data. Int. J. Cardiol..

[56] Sorgente A., Chierchia G.B., De Asmundis C., Sarkozy A., Namdar M., Capulzini L., Yazaki Y., Mller-Burri S.A., Bayrak F., Brugada P. (2011). Pulmonary Vein Ostium Shape and Orientation as Possible Predictors of Occlusion in Patients with Drug-Refractory Paroxysmal Atrial Fibrillation Undergoing Cryoballoon Ablation. Europace.

[57] Nedios S., Tang M., Roser M., Solowjowa N., Gerds-Li J.H., Fleck E., Kriatselis C. (2011). Characteristic Changes of Volume and Three-Dimensional Structure of the Left Atrium in Different Forms of Atrial Fibrillation: Predictive Value after Ablative Treatment. J. Interv. Card. Electrophysiol..

[58] Nedios S., Koutalas E., Sommer P., Arya A., Rolf S., Husser D., Bollmann A., Hindricks G., Breithardt O. (2017). Asymmetrical Left Atrial Remodelling in Atrial Fibrillation: Relation with Diastolic Dysfunction and Long-Term Ablation Outcomes. Europace.

[59] Nedios S., Löbe S., Knopp H., Seewöster T., Heijman J., Crijns H.J.G.M., Arya A., Bollmann A., Hindricks G., Dinov B. (2021). Left Atrial Activation and Asymmetric Anatomical Remodeling in Patients with Atrial Fibrillation: The Relation between Anatomy and Function. Clin. Cardiol..

[60] Guo F., Li C., Yang L., Chen C., Chen Y., Ni J., Fu R., Jiao Y., Meng Y. (2021). Impact of Left Atrial Geometric Remodeling on Late Atrial Fibrillation Recurrence after Catheter Ablation. J. Cardiovasc. Med..

[61] Bieging E.T., McGann C.J., Morris A., Rassa A., Cates J. (2014). Left Atrial Spherical Shape Change in Atrial Fibrillation. J. Cardiovasc. Magn. Reson..

[62] Bisbal F., Gomez-Pulido F., Cabanas-Grandio P., Akoum N., Calvo M., Andreu D., Prat-Gonzalez S., Perea R.J., Villuendas R., Berruezo A. (2016). Left Atrial Geometry Improves Risk Prediction of Thromboembolic Events in Patients with Atrial Fibrillation. J. Cardiovasc. Electrophysiol..

[63] Osmanagic A., Möller S., Osmanagic A., Sheta H.M., Vinther K.H., Egstrup K. (2016). Left Atrial Sphericity Index Predicts Early Recurrence of Atrial Fibrillation After Direct-Current Cardioversion: An Echocardiographic Study. Clin. Cardiol..

[64] Nakamori S., Ngo L.H., Tugal D., Manning W.J., Nezafat R. (2018). Incremental Value of Left Atrial Geometric Remodeling in Predicting Late Atrial Fibrillation Recurrence after Pulmonary Vein Isolation: A Cardiovascular Magnetic Resonance Study. J. Am. Heart Assoc..

[65] Bisbal F., Alarcón F., Ferrero-De-Loma-Osorio A., González-Ferrer J.J., Alonso C., Pachón M., Tizón H., Cabanas-Grandío P., Sanchez M., Benito E. (2018). Left Atrial Geometry and Outcome of Atrial Fibrillation Ablation: Results from the Multicentre LAGO-AF Study. Eur. Heart J. Cardiovasc. Imaging.

[66] Bisbal F., Guiu E., Cabanas P., Calvo N., Berruezo A., Tolosana J.M., Arbelo E., Vidal B., De Caralt T.M., Sitges M. (2014). Reversal of Spherical Remodelling of the Left Atrium after Pulmonary Vein Isolation: Incidence and Predictors. Europace.

[67] Mulder M.J., Kemme M.J.B., Visser C.L., Hopman L.H.G.A., van Diemen P.A., van de Ven P.M., Götte M.J.W., Danad I., Knaapen P., van Rossum A.C. (2020). Left Atrial Sphericity as a Marker of Atrial Remodeling: Comparison of Atrial Fibrillation Patients and Controls. Int. J. Cardiol..

[68] Bossard M., Knecht S., Aeschbacher S., Buechel R.R., Hochgruber T., Zimmermann A.J., Kessel-Schaefer A., Stephan F.P., VÖllmin G., Pradella M. (2017). Conventional versus 3-D Echocardiography to Predict Arrhythmia Recurrence after Atrial Fibrillation Ablation. J. Cardiovasc. Electrophysiol..

[69] Lamata P. (2021). Unleashing the Prognostic Value of Atrial Shape in Atrial Fibrillation. Heart Rhythm O2.

[70] Bieging E.T., Morris A., Wilson B.D., McGann C.J., Marrouche N.F., Cates J. (2018). Left Atrial Shape Predicts Recurrence after Atrial Fibrillation Catheter Ablation. J. Cardiovasc. Electrophysiol..

[71] Goparaju A., Csecs I., Morris A., Kholmovski E., Marrouche N., Whitaker R., Elhabian S. (2018). On the Evaluation and Validation of Off-the-Shelf Statistical Shape Modeling Tools: A Clinical Application. Shape Med. Imaging.

[72] Goparaju A., Iyer K., Bône A., Hu N., Henninger H.B., Anderson A.E., Durrleman S., Jacxsens M., Morris A., Csecs I. (2022). Benchmarking Off-the-Shelf Statistical Shape Modeling Tools in Clinical Applications. Med. Image Anal..

[73] Ambellan F., Lamecker H., von Tycowicz C., Zachow S. (2019). Statistical Shape Models: Understanding and Mastering Variation in Anatomy. Biomedical Visualisation.

[74] Nagel C., Schuler S., Dössel O., Loewe A. (2021). A Bi-Atrial Statistical Shape Model for Large-Scale in Silico Studies of Human Atria: Model Development and Application to ECG Simulations. Med. Image Anal..

[75] Adams J., Khan N., Morris A., Elhabian S. (2022). Spatiotemporal Cardiac Statistical Shape Modeling: A Data-Driven Approach. Stat. Atlases Comput. Models Heart.

[76] Iyer K., Elhabian S. Mesh2SSM: From Surface Meshes to Statistical Shape Models of Anatomy. Proceedings of the International Conference on Medical Image Computing and Computer-Assisted Intervention.

[77] Cates J., Bieging E., Morris A., Gardner G., Akoum N., Kholmovski E., Marrouche N., McGann C., Macleod R.S. (2015). Computational Shape Models Characterize Shape Change of the Left Atrium in Atrial Fibrillation. Clin. Med. Insights Cardiol..

[78] Cootes T.F., Taylor C.J., Cooper D.H., Graham J. (1995). Active Shape Models—Their Training and Application. Comput. Vis. Image Underst..

[79] Cates J., Fletcher P.T., Styner M., Shenton M., Whitaker R. Shape Modeling and Analysis with Entropy-Based Particle Systems. Proceedings of the Information Processing in Medical Imaging: 20th International Conference, IPMI 2007.

[80] Greenacre M., Groenen P.J.F., Hastie T., D’Enza A.I., Markos A., Tuzhilina E. (2022). Principal Component Analysis. Nat. Rev. Methods Primers.

[81] Jollife I.T., Cadima J. (2016). Principal Component Analysis: A Review and Recent Developments. Philos. Trans. R. Soc. A Math. Phys. Eng. Sci..

[82] Banerjee A., Camps J., Zacur E., Andrews C.M., Rudy Y., Choudhury R.P., Rodriguez B., Grau V. (2021). A Completely Automated Pipeline for 3D Reconstruction of Human Heart from 2D Cine Magnetic Resonance Slices. Philos. Trans. R. Soc. A Math. Phys. Eng. Sci..

[83] Banerjee A., Zacur E., Choudhury R.P., Grau V. Optimised Misalignment Correction from Cine MR Slices Using Statistical Shape Model. Proceedings of the Annual Conference on Medical Image Understanding and Analysis.

[84] Piazzese C., Carminati M.C., Pepi M., Caiani E.G. (2017). Statistical Shape Models of the Heart: Applications to Cardiac Imaging. Statistical Shape and Deformation Analysis: Methods, Implementation and Applications.

[85] Bhalodia R., Subramanian A., Morris A., Cates J., Whitaker R., Kholmovski E., Marrouche N., Elhabian S. (2019). Does Alignment in Statistical Shape Modeling of Left Atrium Appendage Impact Stroke Prediction?. Comput. Cardiol..

[86] Jia S., Nivet H., Harrison J., Pennec X., Camaioni C., Jais P., Cochet H., Sermesant M. (2021). Left Atrial Shape Is Independent Predictor of Arrhythmia Recurrence after Catheter Ablation for Atrial Fibrillation: A Shape Statistics Study. Heart Rhythm O2.

[87] Varela M., Bisbal F., Zacur E., Berruezo A., Aslanidi O.V., Mont L., Lamata P. (2017). Novel Computational Analysis of Left Atrial Anatomy Improves Prediction of Atrial Fibrillation Recurrence after Ablation. Front. Physiol..

[88] Nairn D., Eichenlaub M., Lehrmann H., Muller-Edenborn B., Chen J., Huang T., Nagel C., Sanchez J., Luongo G., Arentz T. (2023). Spatial Correlation of Left Atrial Low Voltage Substrate in Sinus Rhythm versus Atrial Fibrillation: Identifying the Pathological Substrate Irrespective of the Rhythm: The Rhythm Specificity of Atrial Low Voltage Substrate. J. Cardiovasc. Electrophysiol..

[89] Labarthe S., Bayer J., Coudière Y., Henry J., Cochet H., Jaïs P., Vigmond E. (2014). A Bilayermodel of Human Atria:Mathematical Background, Construction, and Assessment. Europace.

[90] Roney C.H., Pashaei A., Meo M., Dubois R., Boyle P.M., Trayanova N.A., Cochet H., Niederer S.A., Vigmond E.J. (2019). Universal Atrial Coordinates Applied to Visualisation, Registration and Construction of Patient Specific Meshes. Med. Image Anal..

[91] Fastl T.E., Tobon-Gomez C., Crozier A., Whitaker J., Rajani R., McCarthy K.P., Sanchez-Quintana D., Ho S.Y., O’Neill M.D., Plank G. (2018). Personalized Computational Modeling of Left Atrial Geometry and Transmural Myofiber Architecture. Med. Image Anal..

[92] Zolotarev A.M., Khan A., Khan R., Slabaugh G., Roney C.H. Predicting Atrial Fibrillation Treatment Outcome with Siamese Multi-Modal Fusion and Cardiac Digital Twins. Proceedings of the Medical Imaging with Deep Learning.

[93] Aronis K.N., Ali R., Trayanova N.A. (2019). The Role of Personalized Atrial Modeling in Understanding Atrial Fibrillation Mechanisms and Improving Treatment. Int. J. Cardiol..

[94] Banerjee A., Zacur E., Choudhury R.P., Grau V. (2022). Automated 3D Whole-Heart Mesh Reconstruction From 2D Cine MR Slices Using Statistical Shape Model. Proceedings of the Annual International Conference of the IEEE Engineering in Medicine and Biology Society.

[95] Li J., Chen K., He L., Luo F., Wang X., Hu Y., Zhao J., Zhu K., Chen X., Zhang Y. (2024). Data-driven Classification of Left Atrial Morphology and Its Predictive Impact on Atrial Fibrillation Catheter Ablation. J. Cardiovasc. Electrophysiol..

[96] Beetz M., Banerjee A., Grau V. (2022). Multi-Domain Variational Autoencoders for Combined Modeling of MRI-Based Biventricular Anatomy and ECG-Based Cardiac Electrophysiology. Front. Physiol..

[97] Beetz M., Banerjee A., Grau V. Multi-Objective Point Cloud Autoencoders for Explainable Myocardial Infarction Prediction. Proceedings of the International Conference on Medical Image Computing and Computer-Assisted Intervention.

[98] Beetz M., Acero J., Banerjee A., Eitel I., Zacur E., Lange T., Stiermaier T., Evertz R., Backhaus S., Thiele H. (2022). Interpretable Cardiac Anatomy Modeling Using Variational Mesh Autoencoders. Front. Cardiovasc. Med..

[99] Muffoletto M., Xu H., Burns R., Suinesiaputra A., Nasopoulou A., Kunze K.P., Neji R., Petersen S.E., Niederer S.A., Rueckert D. (2024). Evaluation of Deep Learning Estimation of Whole Heart Anatomy from Automated Cardiovascular Magnetic Resonance Short- and Long-Axis Analyses in UK Biobank. Eur. Heart J. Cardiovasc. Imaging.

[100] Beetz M., Banerjee A., Grau V. (2021). Generating Subpopulation-Specific Biventricular Anatomy Models Using Conditional Point Cloud Variational Autoencoders. Proceedings of the International Workshop on Statistical Atlases and Computational Models of the Heart.

[101] Peng J., Beetz M., Banerjee A., Chen M., Grau V. Generating Virtual Populations of 3D Cardiac Anatomies with Snowflake-Net. Proceedings of the International Workshop on Statistical Atlases and Computational Models of the Heart.

[102] Adams J., Khan N., Morris A., Elhabian S. (2023). Learning Spatiotemporal Statistical Shape Models for Non-Linear Dynamic Anatomies. Front. Bioeng. Biotechnol..

[103] Beetz M., Banerjee A., Grau V. (2024). Modeling 3D Cardiac Contraction and Relaxation With Point Cloud Deformation Networks. IEEE J. Biomed. Health Inform..

[104] Beetz M., Acero J.C., Banerjee A., Eitel I., Zacur E., Lange T., Stiermaier T., Evertz R., Backhaus S.J., Thiele H. Mesh U-Nets for 3D Cardiac Deformation Modeling. Proceedings of the International Workshop on Statistical Atlases and Computational Models of the Heart.

[105] Sanatkhani S., Nedios S., Menon P.G., Bollmann A., Hindricks G., Shroff S.G. (2021). Subject-Specific Calculation of Left Atrial Appendage Blood-Borne Particle Residence Time Distribution in Atrial Fibrillation. Front. Physiol..

[106] Garcia-Isla G., Olivares A.L., Silva E., Nunez-Garcia M., Butakoff C., Sanchez-Quintana D., Morales G.H., Freixa X., Noailly J., De Potter T. (2018). Sensitivity Analysis of Geometrical Parameters to Study Haemodynamics and Thrombus Formation in the Left Atrial Appendage. Int. J. Numer. Method. Biomed. Eng..

